# User Engagement Clusters of an 8-Week Digital Mental Health Intervention Guided by a Relational Agent (Woebot): Exploratory Study

**DOI:** 10.2196/47198

**Published:** 2023-10-13

**Authors:** Valerie Hoffman, Megan Flom, Timothy Y Mariano, Emil Chiauzzi, Andre Williams, Andrew Kirvin-Quamme, Sarah Pajarito, Emily Durden, Olga Perski

**Affiliations:** 1 Woebot Health, Inc. San Francisco, CA United States; 2 Rehabilitation Research & Development Service Center for Neurorestoration and Neurotechnology Department of Veterans Affairs Providence Healthcare System Providence, RI United States; 3 Department of Psychiatry and Human Behavior Warren Alpert Medical School of Brown University Providence, RI United States; 4 Herbert Wertheim School of Public Health and Human Longevity Science University of California San Diego, CA United States; 5 Faculty of Social Sciences Tampere University Tampere Finland

**Keywords:** anxiety, clustering, depression, digital health, digital mental health intervention, mental health, relational agents, user engagement

## Abstract

**Background:**

With the proliferation of digital mental health interventions (DMHIs) guided by relational agents, little is known about the behavioral, cognitive, and affective engagement components associated with symptom improvement over time. Obtaining a better understanding could lend clues about recommended use for particular subgroups of the population, the potency of different intervention components, and the mechanisms underlying the intervention’s success.

**Objective:**

This exploratory study applied clustering techniques to a range of engagement indicators, which were mapped to the intervention’s active components and the connect, attend, participate, and enact (CAPE) model, to examine the prevalence and characterization of each identified cluster among users of a relational agent-guided DMHI.

**Methods:**

We invited adults aged 18 years or older who were interested in using digital support to help with mood management or stress reduction through social media to participate in an 8-week DMHI guided by a natural language processing–supported relational agent, Woebot. Users completed assessments of affective and cognitive engagement, working alliance as measured by goal and task working alliance subscale scores, and enactment (ie, application of therapeutic recommendations in real-world settings). The app passively collected data on behavioral engagement (ie, utilization). We applied agglomerative hierarchical clustering analysis to the engagement indicators to identify the number of clusters that provided the best fit to the data collected, characterized the clusters, and then examined associations with baseline demographic and clinical characteristics as well as mental health outcomes at week 8.

**Results:**

Exploratory analyses (n=202) supported 3 clusters: (1) “typical utilizers” (n=81, 40%), who had intermediate levels of behavioral engagement; (2) “early utilizers” (n=58, 29%), who had the nominally highest levels of behavioral engagement in week 1; and (3) “efficient engagers” (n=63, 31%), who had significantly higher levels of affective and cognitive engagement but the lowest level of behavioral engagement. With respect to mental health baseline and outcome measures, efficient engagers had significantly higher levels of baseline resilience (*P*<.001) and greater declines in depressive symptoms (*P*=.01) and stress (*P*=.01) from baseline to week 8 compared to typical utilizers. Significant differences across clusters were found by age, gender identity, race and ethnicity, sexual orientation, education, and insurance coverage. The main analytic findings remained robust in sensitivity analyses.

**Conclusions:**

There were 3 distinct engagement clusters found, each with distinct baseline demographic and clinical traits and mental health outcomes. Additional research is needed to inform fine-grained recommendations regarding optimal engagement and to determine the best sequence of particular intervention components with known potency. The findings represent an important first step in disentangling the complex interplay between different affective, cognitive, and behavioral engagement indicators and outcomes associated with use of a DMHI incorporating a natural language processing–supported relational agent.

**Trial Registration:**

ClinicalTrials.gov NCT05672745; https://classic.clinicaltrials.gov/ct2/show/NCT05672745

## Introduction

### Overview

Mental health problems continue to increase in prevalence despite the availability of effective treatment [1]. In 2019, over 1 in 5 American adults had any mental illness in the past year [1], with an additional number with subthreshold levels manifested as stress or burnout [2,3]. Since then, the world has experienced the global COVID-19 pandemic, which is reported to be responsible for a worldwide 25% increase in anxiety and depression [4]. Because of the variance in individual responses to these mental health challenges, it is critical to use strategies that may improve resilience in vulnerable individuals [5].

### The Move to Digital Mental Health Interventions and Understanding Engagement

Amplified by pandemic-related widespread lockdowns and other restrictions, the undiminished need for mental health services has trended toward increased uptake of web-based psychotherapeutic interventions [6], also known as digital mental health interventions (DMHIs). Access issues seem to have been a major driver of this trend. In-person interventions have traditionally suffered from structural access barriers such as distance from a provider, transportation difficulties, scheduling, childcare issues, and stigma [1]. DMHIs offer ease of access despite stigma, 24-7 availability, and potential application of evidence-based treatments with fidelity at the time of need [7]. These developments, combined with the rapid growth of DMHIs, have raised concerns about the efficacy of such interventions, but meta-analyses have found equivalent efficacy [8] when comparing DMHIs to face-to-face treatments such as cognitive behavioral therapy (CBT). DMHIs delivered through smartphone apps have demonstrated moderate effect sizes for both depression and anxiety outcomes [9,10].

Despite their documented benefits, real-world engagement (ie, a multifaceted construct with behavioral, cognitive, and affective elements) [11] with DMHIs has been suboptimal. For example, a study using a systematic search of popular mental health apps found a median of 4% of users opening the app on any given day [12]. Reported barriers to engagement with DMHIs include lack of personalization, the presence of more severe mental health symptoms, and technological issues with the app [13]. These findings are considered important, as it is assumed that higher engagement leads to improved mental health outcomes, although this pattern has not been consistently observed in the literature [14]. While some studies report mostly significant associations between overall app use, such as time spent in the app or times accessing the app, and mental health outcomes [15,16], other studies have found no significant associations [17,18]. Several DMHI studies have even found significant relationships between lower levels of engagement and favorable mental health outcomes [17,19]. Although these analyses had different methodologies and variable adjustments that preclude direct comparisons across study findings, they may be interpreted to suggest that more engagement is not always better. For example, it has been hypothesized that the types of intervention components accessed [20] and the order of access may be more important for improved outcomes [21].

Researchers therefore continue to explore the impact of engagement on mental health outcomes with a view to determining optimal use recommendations (eg, following experimental work informed by initial observations). Several studies have used machine learning [22] or cluster analysis [23,24] techniques to define subtypes of user engagement patterns that relate to mental health outcomes. For example, a study by Chien and colleagues [22] used a machine learning technique to differentiate DMHI users through several behavioral engagement indicators passively collected during the 14-week intervention. This study identified user engagement patterns based on the extent of use, speed and extent of disengagement, and timing of engagement. Comparisons of anxiety and depression outcomes across user clusters revealed several important insights. First, despite an initial assumption that higher levels of engagement are linearly associated with greater symptomatic improvement, this study found that those labeled as “high engagers with rapid disengagement” had the greatest mean decreases in Patient Health Questionnaire (PHQ)-9 item depressive symptoms at the end of the intervention, followed by the “highest engagers” (the other 3 clusters included characterizations as “low engagers,” “late engagers,” and “high engagers with moderate decrease”). All groups, however, had significant improvements over the course of the intervention. Unfortunately, there may be multiple routes to successful outcomes. One study found a link between overall engagement and more rapid symptom improvement with a DMHI, although users with different patterns of engagement all achieved comparable symptomatic (PHQ-9 and Generalized Anxiety Disorder-7 item scale [GAD-7]) improvement over the course of intervention [23].

### Modeling User Engagement Patterns

The association between engagement and outcomes, and thus optimal use recommendations, also might vary by demographic and clinical characteristics, given differences found in engagement in general. For example, women tend to have higher levels of engagement with DMHIs [13,25] as do older adults, those with higher educational attainment, those who work full-time, and those in a relationship. Those with greater severity of baseline symptoms, conversely, tend to have lower engagement levels [13,26]. Understanding these differential patterns and ensuring that DMHI engagement models address them could improve the ability to tailor recommendations to particular patient subgroups, which in turn may help improve population-level outcomes.

### The Connect, Attend, Participate, and Enact Model

In general, models of user engagement patterns are only as good as the variables that comprise them. Thus, researchers continue to consider new theoretical models of engagement, as well as a wider array of both passively and actively collected objective and subjective engagement indicators [18,27] that capture a user’s degree of interest, affect, attention, and preferences [11]. For example, Piotrowska and colleagues [28] studied parent engagement patterns in child mental health programs to create the connect, attend, participate, and enact (CAPE) model. This model considers behavioral, cognitive, and affective engagement indicators, from the level of interest in an intervention among those eligible (connect), to having a continuous presence in the intervention (attend), to actively engaging with the intervention content (participate), and finally to using knowledge or strategies learned during the intervention in daily life (enact). A systematic review applied the CAPE framework to evaluate digital mental health and well-being programs for perinatal women [29], with findings indicating that few studies of engagement report enactment, despite its importance as an indicator of content assimilation. The value of enactment, however, is not a new concept. Studies of psychotherapy have found that psychotherapy attendance is not a reliable indicator of engagement and that efforts to make changes within and between sessions (eg, homework completion) represent a more meaningful measure [30]. Integrating enactment (ie, cognitive and behavioral engagement) into a DMHI analytical model could provide a more comprehensive picture of engagement, although such measures tend to be difficult to collect within typical DMHI user experiences.

### Working Alliance

Using an integrated approach focused on both objective utilization and subjective engagement metrics might be a particularly important application for DMHIs that incorporate working alliance [31] into the care structure. Bordin [32] defined a working alliance as encompassing 3 constructs representing the relationship between the therapist and client: (1) goal setting, (2) agreement on how to accomplish the goal (task setting), and (3) the development of a personal bond. The use of working alliance subscales as measures of engagement furthermore closely aligns with the supportive accountability model (ie, the user being responsible for answering their coach or therapist). The creators of this model, Mohr and colleagues [33], describe supportive accountability as the force driving the science behind how long a user utilizes or engages with an intervention. Similarly, we argue the closely related concept of working alliance is one of the forces driving the science behind DMHI engagement (with enactment being another important aspect of engagement that likely drives positive outcomes).

### Relational Agents

The creation of relational agents, otherwise known as conversational agents or chatbots, has provided an innovative mental health care pathway within the class of DMHIs to extend the efficacy of face-to-face therapies. Relational agents typically guide the user through different components of the intervention, including psychoeducation, CBT-based exercises, mood tracking, journaling, and real-time conversational interactions with the agent itself. Relational agents have shown promising preliminary efficacy findings [34,35] and even demonstrated the capability of the user forming a working alliance with the technology [36,37]. The strong associations between working alliance [32] and mental health outcomes found in previous literature [38] could potentially extend to relational agents as well. If they do, the supportive accountability [39] provided by a therapist or, in this case, the relational agent itself may represent a unique engagement-promoting strategy not previously conceptualized as such [40].

Few studies have investigated the engagement patterns of relational agents, much less how patterns of use of specific components of the intervention relate to favorable outcomes [6]. This is especially important because interventions guided by relational agents tend to incorporate a complex interplay of components available for user access that likely range in potency (ie, the power to influence behavior) and therapeutic value (eg, mood tracking, psychoeducation, interactions with the agent, and CBT exercises). In addition to obscuring the mechanistic underpinnings of the active parts of the intervention, this lack of knowledge about how engagement relates to outcomes precludes scientifically-grounded use recommendations and adherence definitions [41].

As a first step toward understanding engagement patterns, demographic or clinical characteristics suggesting distinctive phenotypes, and associated mental health outcomes, we designed an exploratory study using data from a previously reported single-arm trial [42]. We examined user engagement with a DHMI, Woebot-LIFE (WB-LIFE), guided by a natural language processing (NLP)–supported relational agent, Woebot, to address the following three aims:

1. Identify the number, prevalence, and characteristics of clusters derived from the analysis of app-measured or self-reported behavioral, cognitive, and affective engagement metrics based on interactions with the intervention components and responses to items that map onto the CAPE theoretical model.
2. Examine differences in baseline demographics and clinical characteristics of users across engagement clusters.
3. Examine whether changes in mental health outcomes such as depressive symptoms, anxiety symptoms, stress, and resilience after 8 weeks of intervention use differ by engagement cluster.

## Methods

### Study Design

Participants in an 8-week, exploratory, single-armed trial of the WB-LIFE DMHI provided data for this study at baseline, and 3 days, 4 weeks, and 8 weeks (ie, end of study assessment) between May 11 and July 20, 2022.

### Recruitment and Eligibility Criteria

Recruitment through social media platform screens yielded 256 enrolled adult participants aged 18 years or older who responded to a social media advertisement to participate in a mental wellness study testing a digital tool used for emotional support and mood management. Inclusion criteria required residence in the United States, owning a smartphone, and having English literacy, while exclusion criteria prohibited those who had used Woebot previously and those with lifetime bipolar disorder, lifetime psychosis (including schizophrenia or schizoaffective disorder), a past-year suicide attempt, or current suicidal ideation with a plan or intent to act. All participants signed informed consent before participating. See Chiauzzi et al [42] for a CONSORT (Consolidated Standards of Reporting Trials) diagram and additional details on recruitment, consent, and remuneration procedures, which totaled up to US $100 over the 8-week study. Approximately 50% (139/256) of the sample had levels of depressive or anxiety symptoms that could be considered clinically elevated (ie, PHQ-8 or GAD-7 of 10 or greater).

### Intervention

WB-LIFE is a DMHI incorporating Woebot, an NLP-supported relational agent that provides an interactional platform to guide users in managing their mood using evidence-based practices such as CBT, interpersonal therapy, and dialectical behavioral therapy. Mood tracking, artificial intelligence–supported text-based conversations driven by the user’s goals, and various tools rooted in evidence-based theoretical constructs and psychoeducational stories can be selected by users. For additional details about the intervention, please refer to Chiauzzi et al [42].

### Measures

#### Behavioral, Cognitive, and Affective Engagement Components

##### Overview

Behavioral, cognitive, and affective engagement measures of interest were selected based on WB-LIFE’s underlying theoretical constructs and in alignment with the CAPE model of engagement outlined above [28]. Models included 39 engagement indicators mapping onto 8 broader groupings (ie, constructs): 32 behavioral engagement indicators passively collected in the app corresponding to 4 different constructs, 6 self-reported affective engagement indicators of 3 constructs, and 1 self-reported cognitive and behavioral engagement indicator of the eighth construct.

##### Behavioral Engagement Constructs

The collected app data included the following four constructs (32 variables) of objective utilization:

Weekly (1-8) sums of the number of days opening the app, which maps to the *A* (attend) of the CAPE model and accounts for the variance not ascribed to the other metrics
2. Weekly (1-8) sums of the number of tools completed, which represents the *P* (participate) of the CAPE model as well as the CBT exercises completed
Weekly (1-8) sums of the number of stories completed, which represents completing psychoeducation components of the intervention
Weekly (1-8) sums of the number of messages exchanged with Woebot, which represents the relational agent connection experienced by the user

Because all study participants were by nature already recruited and enrolled in the study, the engagement model could not include the *C* (connect) of the CAPE model.

##### Affective Engagement Constructs

Study participants actively provided self-report data to quantify 6 variables representing 3 subjective measures of affective engagement. The factor structure of the 12-item Working Alliance Inventory-Short Report (WAI-SR) represents 3 subscales (goal, task, and bond) that map onto Bordin’s [32] theory of working alliance. The word *therapist* was changed in the assessment to *Woebot* to enable the user to rate the working alliance felt with the relational agent itself. These data provided 6 variables representing three constructs in our utilization and engagement models, as follows:

1. goal subscale scores at 3 days and 8 weeks to capture user ratings of the agreement between the user and Woebot on the goals of the intervention,
2. task subscale score at 3 days and 8 weeks to measure user ratings of the agreement between the user and Woebot on the plan to accomplish the goals, and
3. bond subscale score at 3 days and 8 weeks to assess the user’s rating of the personal bond felt with Woebot.

##### Cognitive and Behavioral Engagement Construct

The final construct representing both cognitive and behavioral aspects of engagement consisted of a single question, asking participants “To what extent did you apply Woebot suggestions in your day to day life?” Responses ranged from “not at all” to “a very large extent” to measure the *E* (enact) of the CAPE model. The responses were transformed to a numeric Likert scale (1-5) for inclusion in the clustering models.

#### Mental Health Outcome Variables

##### Overview

Analyses focused on 4 mental health outcome variables of interest. Because only about half of the sample had clinically significant levels of depressive or anxiety symptoms at baseline, 2 more general measures of mental health wellness were selected for analysis: stress and resilience. Baseline variables were examined as clinical characteristics and compared across clusters; change scores of each mental health outcome between baseline and week 8 were compared across clusters as a preliminary, exploratory analysis.

##### PHQ-8 Outcome Variable

The PHQ-8 contains 8 self-reported variables that measure depressive symptoms experienced in the past 2 weeks [43]. Response options range from “not at all” (0) to “nearly every day” (3). Scores range from 0 to 24, with severity cutoffs of 0-4 (less than mild), 5-9 (mild), 10-14 (moderate), 15-19 (moderately severe), and 20-24 (severe) [44]. A score of 10 is a reasonable cutoff point for major depression, having good sensitivity and specificity [44]. Analyses included PHQ-8 scores at baseline and week 8 (end of intervention).

##### GAD-7 Outcome Variable

The GAD-7 contains 7 self-reported items that assess anxiety symptoms experienced in the past 2 weeks [45]. Response options range from “not at all” (0) to “nearly every day” (3). Scores range from 0 to 21 with severity cutoffs of 0-4 (less than mild), 5-9 (mild), 10-14 (moderate), and 15-21 (severe). A score of 10 is a reasonable cutoff point for generalized anxiety disorder [45]. Analyses included GAD-7 scores at baseline and week 8 (end of intervention).

##### Perceived Stress Scale Outcome Variable

Study participants completed the Perceived Stress Scale (PSS) at baseline and 8 weeks as a way to measure how often they felt life was unpredictable, uncontrollable, and overwhelming in the past month [46]. The PSS contains 10 items with responses that range from “never” (0) to “very often” (4), with the summed scale scores ranging from 0 to 40. PSS score categorizations include low perceived stress (0-13), moderate stress (14-26), and high stress (27-40).

##### Brief Resilience Scale Outcome Variable

The Brief Resilience Scale (BRS) includes 6 items to assess an individual’s perceived ability to bounce back or recover from stress or a setback [47]. The 6 items contain statements about the user’s typical responses to stressful events, with respondents indicating the extent to which they agree with each statement using a 5-point Likert scale ranging from 1 (strongly disagree) to 5 (strongly agree). The 3 items that are negatively framed are reverse-coded, and all items are summed and divided by 6 to obtain the overall BRS score.

#### Baseline Demographic and Clinical Characteristics

Baseline assessments contained questions regarding the age of the participant, gender identity, sexual orientation, race and ethnicity, education, employment, marital status, insurance coverage, and concurrent mental health treatment (defined as currently seeing a therapist or taking medication for a psychiatric condition at either the baseline or 8-week assessment). Average baseline values for each mental health outcome of interest were also compared across clusters.

### Data Analysis

Analyses were conducted in R (version 4.2.2; R Core Team) using the *cluster* package [48]. The code is available upon request from the corresponding author. All variables were centered and scaled to a mean of 0 and SD of 1 before clustering.

Agglomerative hierarchical cluster analysis was run by entering the 39 engagement variables described above to determine if meaningful groups could be detected [49]. Agglomerative hierarchical clustering can be considered an unsupervised learning algorithm that initially treats each object as a single cluster and, at each step of the algorithm, clusters that are most similar are combined into a new cluster. This procedure is repeated until all objects (or data points) are members of one single cluster. To ensure the rigor of the findings, 2 distinct approaches were used to determine the number of clusters to retain: the elbow method and the gap statistic. Multiple seeds were also run to be certain that the identified clustering pattern was stable [50,51]. A total of 54 (21.1%) participants were dropped from clustering analysis due to at least one missing data point over the 8-week period. Sensitivity analyses were also conducted using different combinations of input variables (eg, only app-measured behavioral variables, given that none were missing from any of the participants) to further assess the extent to which the clustering patterns were stable and robust. For the primary model as well as sensitivity models, the Ward [52] linkage method was used and presented given that it best handles noisy data; however, all models were also conducted using the average linkage method (the default for agglomerative hierarchical clustering in R), and the exact same pattern of results was found. The agglomerative coefficient for both methods for all models was always greater than 0.98.

The characterization and naming of each cluster required an examination of the cognitive, behavioral, and affective engagement variables across each cluster. ANOVA compared mean utilization and engagement scores across clusters, with α set to .05. The Fisher exact test was used to compare proportions of participants in each cluster who responded to each of the 5 response categories of the enactment question.

We did not have sufficient power to assess baseline predictors of clustering assignment using multinomial logistic regression; however, we examined baseline differences in clinical and demographic characteristics by cluster using chi-square, Fisher exact, and ANOVA tests, as appropriate. Given the exploratory nature of these analyses, we did not account for multiple comparisons other than in post hoc pairwise comparisons, for which *P* values were adjusted using the Holm correction.

### Ethics Approval

Western International Review Board-Copernicus Group approved the study protocol on January 20, 2022 (#20216751). This study was an additional, exploratory analysis of the sample collected for and reported in Chiauzzi et al [42], which was retrospectively registered on ClinicalTrials.gov (NCT05672745) on January 5, 2023.

## Results

### Cluster Prevalence and Characteristics

**Figure 1:**
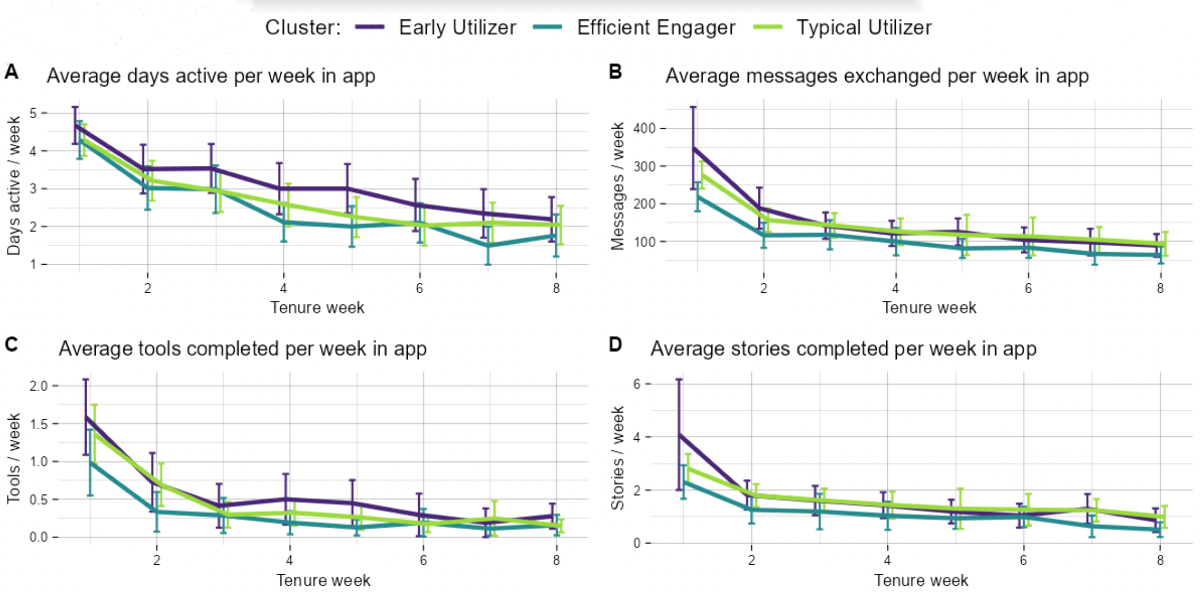
Utilization metrics by cluster (average metric with SE bars plotted for each tenure week for 8 weeks of intervention). A: Average days active per week in app; B: Average messages exchanged per week in app; C: Average tools completed per week in app; D: Average stories completed per week in app.

**Figure 2:**
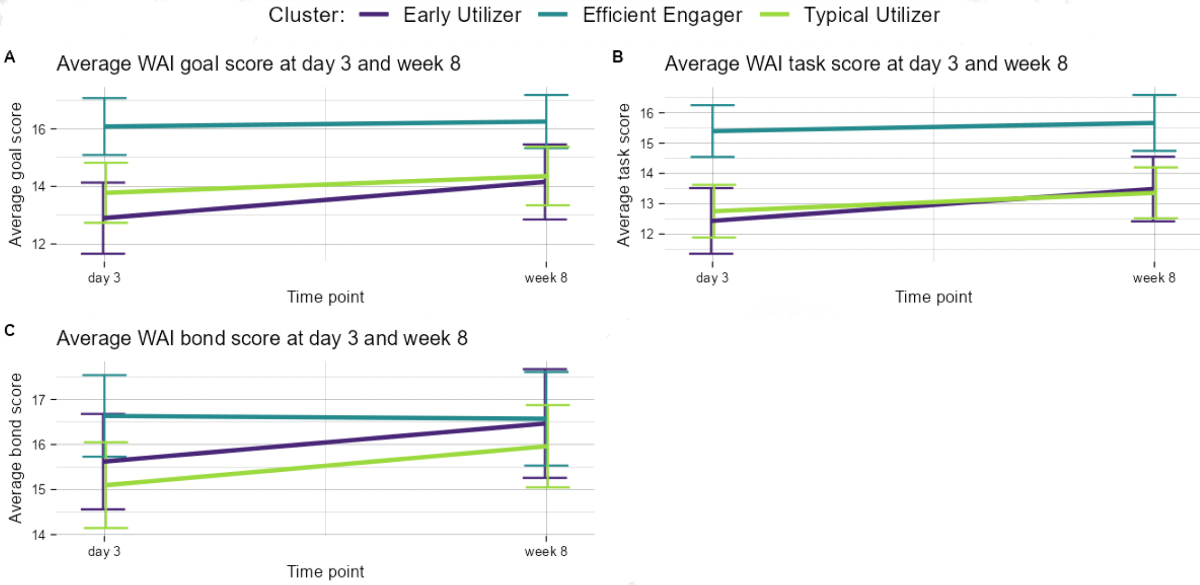
Therapeutic alliance engagement metrics by cluster (average metric with SE bars for day 3 and week 8). WAI: Working Alliance Inventory. A: Average WAI goal score at day 3 and week 8; B: Average WAI task score at day 3 and week 8; C: Average WAI bond score at day 3 and week 8.

A total of 3 clusters were identified and named “typical utilizers,” “early utilizers,” and “efficient engagers.” Figure 1 shows the 8-week behavioral variables measured in-app across the clusters, and Figure 2 shows the affective and behavioral self-reported engagement variables by cluster (Table S1 in Multimedia Appendix 1 shows the data in tabular form). Table 1 shows the frequencies of the cognitive and behavioral engagement metric, enactment, measured at week 8. Although error bars were overlapping (thus indicating nonsignificant differences in behavioral engagement), typical utilizers (81/202, 40%) were the largest cluster, and had midlevels of behavioral engagement (Figure 1) and the lowest levels of therapeutic alliance and enactment (Figure 2). Early utilizers (58/202, 29%) initially had high levels of behavioral engagement that tapered off to levels very similar to typical utilizers after 2 weeks and lower alliance and enactment to efficient engagers (similar to typical utilizers). Efficient engagers (63/202, 31%) had the lowest behavioral engagement measures but a statistically higher therapeutic alliance at baseline and 8 weeks than users in the other 2 clusters (see nonoverlapping error bars in Figure 2). Enactment also significantly differed across the clusters, with post hoc pairwise tests indicating a difference between efficient engagers and the other 2 clusters (efficient engagers vs typical utilizers, *P*<.001; efficient engagers vs early utilizers, *P*=.003). Specifically, efficient engagers reported applying what they learned outside of the app more than others.

Sensitivity analyses that incorporated only the behavioral engagement variables into the clustering analyses identified the same 3 clusters and are presented in Multimedia Appendix 1. In our sample of 256 users, 54 (21.1%) had missing data on at least one of the subjective measures entered into the analysis, precluding the analysis of engagement patterns for a portion of the sample. Nevertheless, similar patterns formed in the sensitivity analysis that focused on clusters of in-app behavioral engagement metrics only, which did include the 54 users with missing engagement data. Furthermore, the 54 users with missing data were uniformly distributed across the 3 clusters.

**Table 1:** Enactment frequencies measured at week 8 for each cluster identified^a^.

To what extent did you apply Woebot suggestions in your day-to-day life?	Typical utilizers (n=81), n (%)	Early utilizers (n=58), n (%)	Efficient engagers (n=63), n (%)
Not at all	2 (2)	2 (3)	2 (3)
Very little	7 (9)	7 (12)	4 (6)
Somewhat	36 (44)	20 (34)	12 (19)
Large extent	22 (27)	20 (34)	22 (35)
Very large extent	14 (17)	9 (16)	23 (37)

^a^*P*=.03 (Fisher exact test).

### Baseline Demographic and Clinical Characterization of Clusters

As seen in Table 2, a greater proportion of efficient engagers were non-Hispanic Black, whereas more non-Hispanic White participants were typical utilizers. Additionally, a greater proportion of efficient engagers identified as male than those in other clusters, and they were nearly 5 years younger. More efficient engagers were heterosexual, reported higher education levels, and had no health insurance (and fewer with private health insurance) than users in other clusters. Stress, anxiety, and depression did not significantly differ at baseline across clusters, although efficient engagers were the only group with an average depressive symptom score within the range indicative of clinical levels of depression (ie, PHQ-8 score ≥10). Baseline resilience was significantly higher in efficient engagers as compared to those in other clusters (post hoc pairwise comparisons with Holm correction: efficient engager vs typical utilizer *P*<.001, efficient engager vs early utilizer *P*=.003, early utilizer vs typical utilizer *P*=.67).

**Table 2:** Baseline sociodemographic and clinical characteristics for users in each cluster.

Baseline characteristic				Typical utilizers (n=81)			Early utilizers (n=58)			Efficient engagers (n=63)			*P* value^a^				Chi-square (*df*)				*F* test (*df*)	
**Sociodemographic characteristics**																						
	Age (years), mean (SD)		39.73 (13.78)			40.60 (13.30)			35.75 (10.28)			.04				N/A^b^				3.22 (2, 127.32)		
	**Race and ethnicity, n (%)**													<.001				24.52 (4)				N/A
		Non-Hispanic Black	13 (16)		8 (14)			26 (41)														
		Non-Hispanic White	42 (52)		40 (69)			31 (49)														
		Other	26 (32)		10 (17)			6 (10)														
	**Gender identity, n (%)**													<.001				14.23 (2)				N/A
		Man	13 (17)		15 (26)			28 (46)														
		Woman	64 (83)		42 (74)			33 (54)														
	**Sexual orientation, n (%)**													.008^c^				N/A				N/A
		Sexual minority	21 (26)		10 (17)			4 (6)														
		Straight or heterosexual	60 (74)		48 (83)			58 (94)														
	**Education, n (%)**													.04				13.27 (6)				N/A
		Graduate or postgraduate degree	16 (21)		19 (33)			29 (47)														
		College degree	33 (43)		22 (39)			21 (34)														
		High school only (grade 9-12)	10 (13)		8 (14)			7 (11)														
		Some college or technical school	18 (23)		8 (14)			5 (8)														
	**Employment, n (%)**													.20				8.58 (6)				N/A
		Employed full-time	36 (47)		32 (56)			41 (66)														
		Employed part-time	9 (12)		5 (9)			8 (13)														
		Not employed	18 (24)		15 (26)			8 (13)														
		Other	13 (17)		5 (9)			5 (8)														
	**Marital status, n (%)**													.29^c^				N/A				N/A
		Divorced, separated, or widowed	9 (12)		7 (13)			2 (3)														
		Married, partnered, or cohabiting	42 (55)		27 (49)			38 (61)														
		Never been married	26 (34)		21 (38)			22 (35)														
	**Insurance, n (%)**													.001^c^				N/A				N/A
		Government insurance	25 (33)		18 (32)			23 (38)														
		Private insurance	44 (58)		35 (62)			20 (33)														
		None or prefer not to answer	7 (9)		3 (5)			18 (30)														
**Clinical characteristics**																						
	Concurrent treatment^d^, n (%)		33 (41)			25 (43)			26 (41)			.96				0.81 (2)				N/A		
	Depressive symptoms (PHQ-8^e^), mean (SD)		8.7 (5.5)			9.4 (5.5)			10.3 (8.0)			.38				N/A				0.97 (2, 122.11)		
	Anxiety symptoms (GAD-7^f^), mean (SD)		8.9 (5.5)			9.2 (5.0)			9.4 (7.4)			.89				N/A				0.12 (2, 124.70)		
	Stress symptoms (PSS^g^), mean (SD)		21.6 (5.0)			21.4 (5.2)			21.8 (6.8)			.93				N/A				0.07 (2, 121.76)		
	Resilience (BRS^h^), mean (SD)		2.8 (0.8)			2.8 (0.7)			3.3 (0.9)			.001				N/A				7.57 (2, 127.14)		

^a^Test for omnibus effect across the 3 clusters.

^b^N/A: not applicable.

^c^Fisher exact test.

^d^Concurrent treatment was defined as any psychotherapy or medication for mental health at any time during the study.

^e^PHQ-8: Patient Health Questionnaire-8 item.

^f^GAD-7: General Anxiety Disorder-7 item scale.

^g^PSS: Perceived Stress Scale.

^h^BRS: Brief Resilience Scale.

### Depressive, Anxiety, Stress, and Resilience Symptom Changes by Clusters

Table 3 reports clinical (ie, depressive and anxiety symptoms) and wellness (ie, stress and resilience) 8-week change scores by cluster. There were significant group differences in depressive symptoms and stress change scores, but not anxiety symptoms or resilience. Post hoc pairwise tests using the Holm correction indicated that efficient engagers had greater reductions in depression and stress as compared to typical utilizers (depressive symptoms: *P*=.01; stress: *P*=.01). Early utilizers did not significantly differ from the other 2 clusters on change in depression (vs typical utilizer *P*=.23; vs efficient engagers *P*=.23) or change in stress (vs typical utilizer *P*=.42; vs efficient engagers *P*=.11).

**Table 3:** Average change in each examined mental health outcome for users in each cluster.

Mental health outcome 8-week change	Typical utilizers (n=81), mean (SD)	Early utilizers (n=58), mean (SD)	Efficient engagers (n=63), mean (SD)	*P* value^a^	*F* test (*df*)
Depressive symptoms (PHQ-8^b^)	–2.4 (5.0)	–4.0 (5.3)	–5.3 (7.1)	.02	4.22 (2, 120.93)
Anxiety symptoms (GAD-7^c^)	–3.4 (5.0)	–4.0 (4.4)	–4.9 (7.4)	.38	0.97 (2, 123.29)
Stress (PSS^d^)	–1.4 (5.5)	–3.1 (7.0)	–4.1 (6.8)	.03	3.54 (2, 119.52)
Resilience (BRS^e^)	0.33 (0.72)	0.43 (0.65)	0.27 (0.79)	.46	0.78 (2, 127.38)

^a^Test for omnibus effect across the 3 clusters.

^b^PHQ-8: Patient Health Questionnaire-8 item.

^c^GAD-7: General Anxiety Disorder-7 item scale.

^d^PSS: Perceived Stress Scale.

^e^BRS: Brief Resilience Scale.

## Discussion

### Principal Results

This exploratory study of behavioral, affective, and cognitive engagement patterns of relational agent users enrolled in a single-arm trial yielded 3 clusters that we labeled “typical utilizers,” “early utilizers,” and “efficient engagers.” Typical utilizers had intermediate levels of behavioral engagement. Early utilizers had the nominally highest levels of initial behavioral engagement measured in-app. The groups were most differentiated by the efficient engagers, who had significantly higher levels of day 3 working alliance as compared to users in the other 2 groups, as well as significantly greater enactment in daily life of concepts learned and practiced in the app.

Several patterns emerged with respect to the demographic and clinical characteristics of study participants that differentiated the clusters. The 3 clusters significantly differed with respect to the proportion of study participants’ age, gender identity, race and ethnicity, sexual orientation, education, and insurance status. Although pairwise comparisons were not done, efficient engagers had the highest proportions of men, non-Hispanic Black, heterosexual, educated, younger, and uninsured, or users not reporting insurance coverage. Because several cells had small sample sizes, these findings should be interpreted with caution until additional investigation can be done with greater numbers of enrolled participants. Finally, besides efficient engagers having higher baseline levels of resilience than users in other clusters, baseline levels of depressive symptoms, anxiety symptoms, or stress did not differ across the clusters. Resilience has long been associated with positive health states [53], acting as a buffer to adversity and helping shield against the formation of mental health issues [54]. Perhaps the resilience combined with the strong formation of working alliance for these individuals worked in the same way to achieve beneficial reductions in depressive symptoms and stress over the course of the intervention as compared to those in other cluster groups.

With respect to mental health outcomes, efficient engagers had significantly greater declines in both depressive symptoms and stress in comparison to typical utilizers. It should be noted, however, that the efficient engagers had the lowest levels of in-app measured behavioral engagement variables, which underscores the importance of not treating these measures alone as a proxy for “good outcomes.” Perhaps efficient engagers were able to get what they needed from the app because they approached the intervention with a clear idea of what they wanted to get out of app interactions (goal) and how they were going to interact with the app to accomplish their goals (task). Beyond the scope of this study but ripe for additional investigation is the determination of specific app components that efficient engagers accessed to “get what they needed,” also referred to as “e-attainment” [23]. The idea of what specific relational agent components are most potent to specific groups (or subgroups) of users is a unique area of inquiry worthy of future study.

### Comparison With Previous Work

This study demonstrated higher than expected levels of behavioral engagement among study participants enrolled in a single-arm trial. For example, in this study, approximately three-quarters of users opened the app in at least 50% of the study weeks (ie, in at least 4 of 8 weeks). In contrast, a review by Fleming et al [55] determined that between 7% and 42% of DHMI app users were categorized as moderate users, as defined by completing between 40% and 60% of modular content or continuing to use the app after 4 weeks. Over half (58%, 148/255) of this study’s participants opened the app on week 8 of the study, which is also much higher than the 0.5%-28.6% of users who completed all modules, the last assessment, or continued to use the app after 6 weeks found in Fleming et al’s [55] review. It should be noted, however, that Fleming et al’s [55] review focused on all DMHI users and not solely those participating in a clinical trial.

Our findings of patterns of engagement among relational agent study participants extend the work presented by several others to include important self-reported measures of affective and cognitive and behavioral engagement that capture aspects of the user’s attention, interest, and affect in an effort to focus on process rather than product [6,56]. For example, the work by Chien and colleagues [22] only included passively detected behavioral engagement indicators when seeking to understand patterns of use most associated with particular sets of outcomes. Our sensitivity analysis that included only app-collected behavioral engagement measures in the clustering yielded weaker results, with less differentiation between clusters, than did our main analysis that included additional self-reported affective and cognitive and behavioral engagement variables, notably goal and task working alliance subscales and enactment. Others have proposed that related cognitive and affective engagement variables defined as usability, likeability, usefulness, and satisfaction are a key part of the mechanism by which engagement affects outcomes [17], and that a comprehensive set of these aspects in combination with behavioral engagement measures (eg, the dynamic, time-varying interplay between behavioral engagement and perceived usefulness) should be examined when quantifying and studying engagement [57]. These views underscore the importance of our inclusion of affective and cognitive engagement aspects such as working alliance and enactment, as well as other behavioral constructs measured in-app, when conducting these types of analyses.

### Strengths and Limitations

To the authors’ knowledge, this study was the first of its kind to investigate patterns of engagement using a comprehensive set of cognitive, behavioral, and affective measures among users of a DMHI guided by an NLP-supported relational agent. We used a rigorous analytical approach assessing the theorized “active ingredients” of the intervention and the previously described CAPE framework. The identified clusters were robust in that they withstood sensitivity analyses. A key finding was that engagement and improvement in outcomes may not be directly proportional to each other, aligning with an emerging body of work in DMHI research that may seem counterintuitive to a traditional approach to mental health treatment that emphasizes adherence to treatment over time to obtain expected benefit. Additional strengths of this investigation were the relative diversity of the sample, high use, and survey completion rates.

The study findings require careful consideration of limitations. First, the study did not have a control group, so any improvement in outcomes might be due to regression to the mean, a digital placebo effect, or spontaneous remission [6,58] rather than the WB-LIFE intervention itself. However, characterizing engagement necessitates exposure to the DMHI in order to conduct the analyses. Second, the full CAPE model [28] could not be tested because all study participants were by nature already connected (*C*) to the intervention with enrollment in the trial. To test this in a future study, one could consider a design in which a clinician determines eligibility for using the DMHI app based on predefined criteria, recommends the app to suitable potential participants, and analyzes the percentage of these referrals that convert to app registrations. Third, in this study, a single question measured enactment (*E*). Additional studies need to include a more specific measure of enactment with items that query the user’s sense of mastery over content learned during the intervention as well as the perceived effectiveness of the enactment itself, potentially at multiple time points. Fourth, the analyses focused on very few sociodemographic or clinical characteristics of potential differentiation across clusters. For example, potentially unmeasured characteristics like previous experience with mental health interventions might have increased the likelihood of setting goals and establishing tasks to accomplish them, which then affected outcomes. The direction of causation cannot be determined from this single-armed trial, so optimal use recommendations might not generalize across populations with varying unmeasured characteristics. Additional research using such approaches as directed acyclic graphs could be used to better inform the direction of causation [59] in studies not incorporating a randomized controlled trial design. Also, given known issues with change score analyses in observational studies [59], the highly exploratory change score analyses pertaining to the association of the engagement clusters with the mental health outcomes must be interpreted with caution. For example, the efficient engagers cluster did have the highest levels of baseline depressive symptoms, which may in part explain the significantly greater magnitude of symptom reduction. Fifth, neither the specific content accessed during app use nor the order of modules accessed, similar to analyses done by Perski et al [60] were examined in this study because of the small sample size. Similar studies with greater numbers of participants would permit more nuanced investigations of this kind. Sixth, the patterns of engagement found might not generalize to users not participating in an incentivized research study. Those in the research study had the presumed intent to utilize the app at the onset of the study for the following 8 weeks, whereas those using the app “in the wild” might have had different expectations at the onset of use. Patterns of engagement likely would be materially different if a health care provider prescribed or recommended WB-LIFE, with past research indicating higher utilization rates with provider oversight or feedback [61]. In addition, the results likely only generalize to those seeking help with mood management or stress reduction with characteristics similar to those in this sample, of whom a majority had clinically elevated depressive or anxiety symptoms.

### Clinical Implications of Findings

The findings, if validated across other data sets and in studies using different experimental designs in future work—ideally randomized controlled trials to test patterns found in this study versus those experienced by users of other types of digital interventions that include longer-term follow-up periods to study the stability of symptom improvements over time—have important implications for recommendations of the frequency, duration, and length of time of optimal use for DMHIs incorporating relational agents to achieve favorable outcomes. This study determined that the best outcomes are not always realized by those with the most in-app measures of behavioral engagement. It is important not to mislabel efficient engagers who achieve substantial symptom reductions but do not participate in long periods of the intervention as “dropouts.” Aspects of engagement that measure attention, interest, and affect, as well as characteristics like baseline resilience, likely matter more than the time spent on using the intervention itself, particularly, perhaps, when using relational agents designed to build and maintain relationships with users. Given the findings of this study, helping users determine goals for the intervention aligned with the capabilities of the intervention and methods to achieve those goals may warrant greater attention, particularly given previous work showing greater consensus and collaboration enhances outcomes [62]. Regarding the CAPE model, connection to the DMHI was not assessed due to the study design; future work characterizing if, how, and when eligible users enroll in a DMHI may help optimize them to individual user needs. Nevertheless, the other constructs of the CAPE model provided a strong theoretical basis to ground the engagement model. Enactment appeared to be a critical concept to cultivate when aiming to facilitate good outcomes among users. Finally, the preliminary exploratory differential associations between engagement and outcomes based on demographic and clinical characteristics underscore the need for a more inclusive conceptualization of engagement—especially concerning DMHIs that use relational agents to deliver mental health care. Efforts to target DMHI apps to the unique needs of groups that do not seem to benefit as completely are necessary for equity, parity, and destigmatization, which underscores the importance of enriching study samples to ensure inclusion of underrepresented populations. The findings also suggest that subgroup membership at or near the onset of the intervention, particularly with respect to goal and task constructs of working alliance, can be used to align the intervention with the needs of the users to ensure the most beneficial outcomes.

### Conclusions

We identified 3 engagement clusters of users of a DMHI incorporating an NLP-supported relational agent that emerged from our preliminary analyses of cognitive, affective, and behavioral engagement metrics. The clusters differed with respect to several mental health outcomes as well as demographic and clinical characteristics. Additional analyses with larger sample sizes and more rigorous study designs are needed to replicate findings among participants of varying baseline levels of symptomatology who seek help with their mood and anxiety, and to inform more fine-grained recommendations regarding optimal use, and to determine the best sequence of specific intervention components for each individual. Nevertheless, the findings represent an important first step in disentangling the complex interplay between aspects of engagement and outcomes associated with the use of a DMHI guided by an NLP-supported relational agent.

## Appendix

Multimedia Appendix 1 Supplemental tables and figures.

## Abbreviations

BRS: Brief Resilience Scale
CAPE: connect, attend, participate, and enact
CBT: cognitive behavioral therapy
CONSORT: Consolidated Standards of Reporting Trials
DMHI: digital mental health intervention
GAD-7: Generalized Anxiety Disorder-7 item scale
NLP: natural language processing
PHQ: Patient Health Questionnaire
PSS: Perceived Stress Scale
WAI-SR: Working Alliance Inventory-Short Report
WB-LIFE: Woebot-LIFE

## Data Availability

The data sets generated during and/or analyzed during this study are available from the corresponding author on reasonable request.
