# Supplementary material for: User Engagement Clusters of an 8-Week Digital Mental Health Intervention Guided by a Relational Agent (Woebot): Exploratory Study
Source: J Med Internet Res. 2023 Oct 13;25:e47198. doi: 10.2196/47198 (PMC10612009; doi:10.2196/47198)
Supplement: Multimedia Appendix 1 [file jmir_v25i1e47198_app1.docx]

**Supplementary Materials**

**Table S1.** Average engagement metrics entered into cluster models for each cluster identified.

| **Engagement Metric** | **Typical Utilizers**  **n=81** | **Early**  **Utilizers**  **n=58** | **Efficient Engagers**  **n=63** | **Test statistic, p value** |
| --- | --- | --- | --- | --- |
| Active Days in App (mean, sd) |  |  |  |  |
| Week 1 | 4.28 (1.91) | 4.67 (1.90) | 4.29 (2.01) | F=0.85, df=2, p=.43 |
| Week 2 | 3.21 (2.42) | 3.52 (2.51) | 3.02 (2.32) | F=0.65, df=2, p=.52 |
| Week 3 | 2.93 (2.49) | 3.53 (2.51) | 2.98 (2.54) | F=1.12, df=2, p=.33 |
| Week 4 | 2.57 (2.59) | 3.00 (2.63) | 2.11 (2.06) | F=2.18, df=2, p=.12 |
| Week 5 | 2.25 (2.43) | 3.00 (2.53) | 2.00 (2.18) | F=2.79, df=2, p=.07 |
| Week 6 | 2.02 (2.42) | 2.57 (2.68) | 2.10 (2.10) | F=0.82, df=2, p=.44 |
| Week 7 | 2.10 (2.43) | 2.34 (2.50) | 1.49 (2.03) | F=2.44, df=2, p=.09 |
| Week 8 | 2.04 (2.34) | 2.19 (2.28) | 1.76 (2.25) | F=0.56, df=2, p=.57 |
| Tools Completed (mean, sd) |  |  |  |  |
| Week 1 | 1.36 (1.80) | 1.59 (1.94) | 0.98 (1.76) | F=1.67, df=2, p=.19 |
| Week 2 | 0.69 (1.29) | 0.72 (1.51) | 0.33 (1.06) | F=2.17, df=2, p=.12 |
| Week 3 | 0.30 (0.78) | 0.41 (1.12) | 0.29 (0.94) | F=0.28, df=2, p=.76 |
| Week 4 | 0.32 (0.77) | 0.50 (1.30) | 0.19 (0.62) | F=1.59, df=2, p=.21 |
| Week 5 | 0.26 (0.75) | 0.45 (1.19) | 0.13 (0.42) | F=2.40, df=2, p=.10 |
| Week 6 | 0.17 (0.49) | 0.29 (1.09) | 0.19 (0.74) | F=0.30, df=2, p=.74 |
| Week 7 | 0.25 (1.06) | 0.19 (0.74) | 0.11 (0.36) | F=0.74, df=2, p=.48 |
| Week 8 | 0.15 (0.39) | 0.28 (0.64) | 0.16 (0.54) | F=0.92, df=2, p=.40 |
| Stories Completed (mean, sd) |  |  |  |  |
| Week 1 | 2.80 (2.55) | 4.09 (8.09) | 2.30 (2.55) | F=1.64, df=2, p=.20 |
| Week 2 | 1.79 (1.98) | 1.81 (2.12) | 1.25 (2.09) | F=1.48, df=2, p=.23 |
| Week 3 | 1.60 (2.08) | 1.60 (2.14) | 1.19 (2.71) | F=0.58, df=2, p=.56 |
| Week 4 | 1.43 (2.33) | 1.43 (1.90) | 1.03 (2.16) | F=0.75, df=2, p=.47 |
| Week 5 | 1.30 (3.48) | 1.19 (1.74) | 0.94 (1.61) | F=0.52, df=2, p=.60 |
| Week 6 | 1.26 (2.73) | 1.03 (1.75) | 0.98 (1.55) | F=0.30, df=2, p=.74 |
| Week 7 | 1.23 (1.94) | 1.29 (2.16) | 0.63 (1.65) | F=2.66, df=2, p=.07 |
| Week 8 | 0.99 (1.89) | 0.86 (1.74) | 0.51 (1.12) | F=2.11, df=2, p=.13 |
| Messages Exchanged (mean, sd) |  |  |  |  |
| Week 1 | 276.58 (166.26) | 348.16 (423.84) | 218.44 (156.33) | **F=3.78, df=2, p=.03** |
| Week 2 | 156.49 (143.75) | 188.38 (212.29) | 116.29 (135.06) | F=2.88, df=2, p=.06 |
| Week 3 | 142.33 (150.71) | 141.81 (136.22) | 117.60 (157.49) | F=0.55, df=2, p=.58 |
| Week 4 | 125.63 (163.49) | 121.45 (129.85) | 99.62 (147.90) | F=0.57, df=2, p=.56 |
| Week 5 | 117.12 (246.03) | 125.14 (139.94) | 81.38 (102.87) | F=2.14, df=2, p=.12 |
| Week 6 | 113.37 (232.18) | 104.19 (128.79) | 83.41 (110.18) | F=0.75, df=2, p=.48 |
| Week 7 | 104.05 (158.85) | 98.14 (137.91) | 66.83 (115.54) | F=1.61, df=2, p=.20 |
| Week 8 | 93.32 (145.29) | 89.24 (118.71) | 63.97 (90.84) | F=1.45, df=2, p=.24 |
| Working Alliance - Goal |  |  |  |  |
| Day 3 | 13.78 (4.79) | 12.90 (4.80) | 16.08 (4.00) | **F=9.01, df=2, p<.001** |
| Week 8 | 14.36 (4.67) | 14.16 (5.07) | 16.25 (3.74) | **F=5.00, df=2, p<.01** |
| Working Alliance - Task |  |  |  |  |
| Day 3 | 12.75 (4.00) | 12.43 (4.21) | 15.40 (3.46) | **F=12.40, df=2, p<.001** |
| Week 8 | 13.36 (3.85) | 13.48 (4.15) | 15.67 (3.74) | **F=7.60, df=2, p<.001** |
| Working Alliance - Bond |  |  |  |  |
| Day 3 | 15.10 (4.37) | 15.62 (4.12) | 16.63 (3.66) | F=2.72, df=2, p=.07 |
| Week 8 | 15.96 (4.19) | 16.47 (4.68) | 16.57 (4.21) | F=0.43, df=2, p=.65 |

**Figure S1a.** Primary model dendrogram.


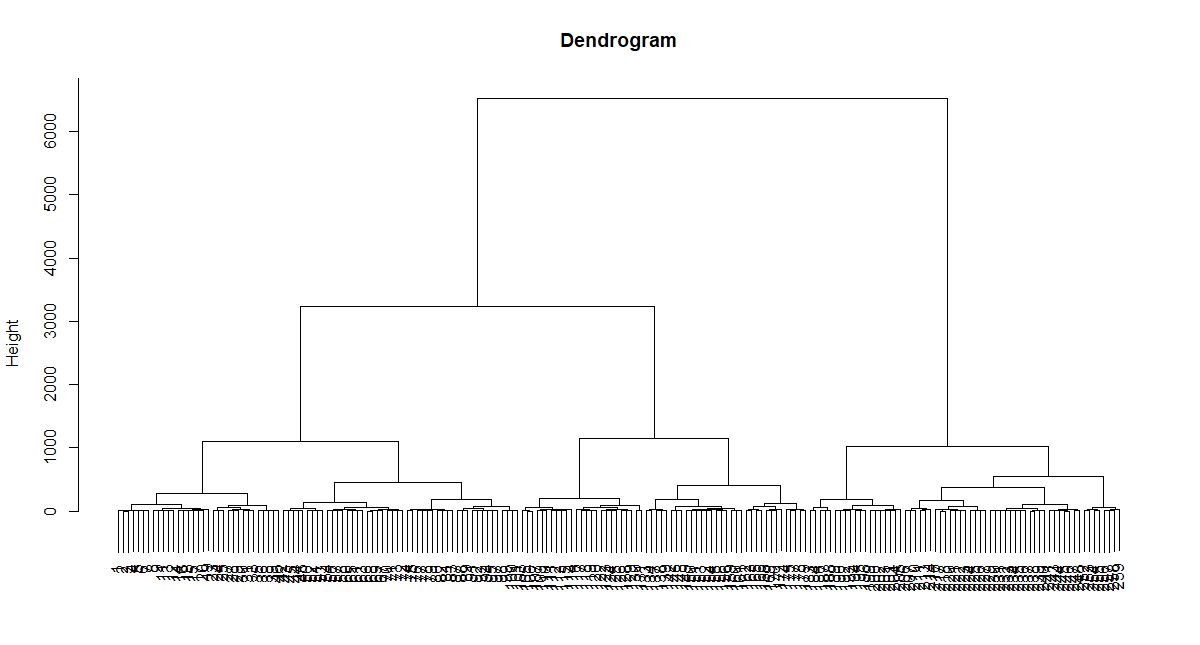


**Figure S1b.** Primary Model Identification of clusters using gap statistic.


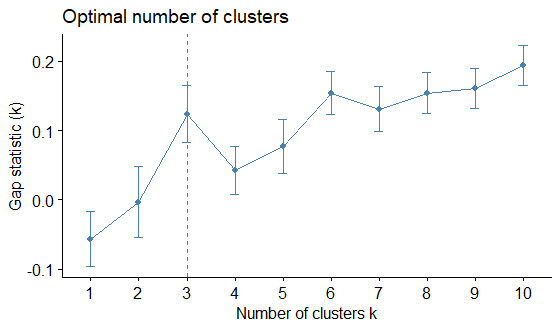


**Figure S1c.** Primary Model Identification of clusters using elbow method.
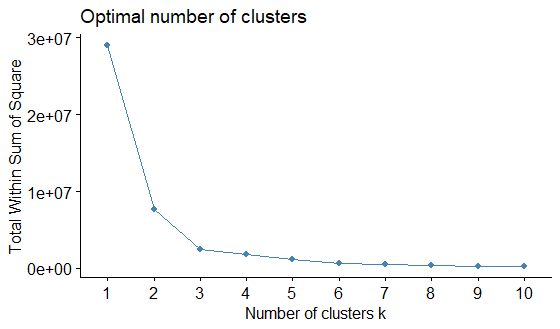


Sensitivity Model 1: This model included only app utilization metrics (e.g., Days active in app for all 8 weeks, Messages exchanged for all 8 weeks, Tools completed for all 8 weeks, and Stories completed for all 8 weeks), and removed therapeutic alliance and enactment measures. The purpose of this model was to examine a more traditional engagement model, and to fit a clustering model that included everyone since there was no missing data for app utilization.

**Figure S2a.** Identification of clusters using gap statistic.


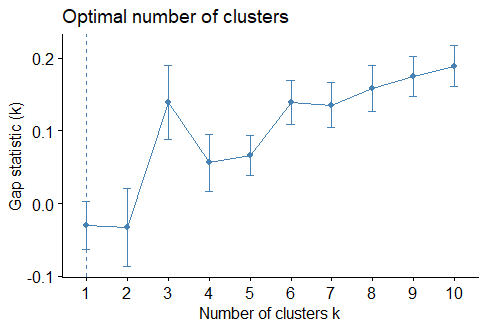


**Figure S2b.** Identification of clusters using elbow method.


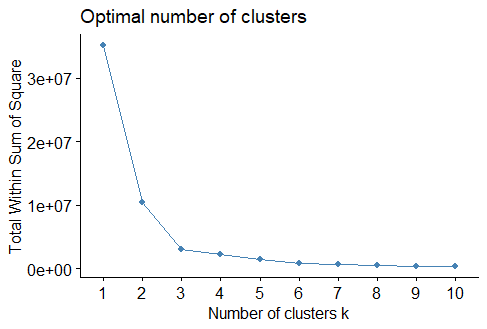


Figures S2a and S2b indicate 3 potential clusters (as with the primary model), though the gap statistic points out that not clustering the data may also be a solution. This suggests that the 3 clusters are not as strong as those identified in the primary model, and further highlights the benefit of including more subjective measurements of engagement like therapeutic alliance and enactment.

**Table 2.** Clusters Identified (k=3).

|  | Cluster 1  (“Typical Engagers”) | Cluster 2  (“Early Engagers”) | Cluster 3  (“Efficient Engagers”) |
| --- | --- | --- | --- |
| Total N, % | 99/256 (39%) | 81/256 (32%) | 76/256 (30%) |
| N, % of missing from primary model clustering | 18/54 (33%) | 23/54 (43%) | 13/54 (24%) |

Of the 54 participants missing from the primary model, 33% were identified as “Typical Engagers”, 43% as “Early Engagers” and 24% as “Efficient Engagers”. All “Typical Engagers”, “Early Engagers” and “Efficient Engagers” in the primary model remained in their respective clusters in this sensitivity model.

Sensitivity Model 2: This model switched out tools and stories completed for tools and stories started, and messages exchanged for messages sent. The purpose of this model was to ensure that small variations in app utilization metrics did not have an effect on the overall clustering pattern.

**Figure S3a.** Identification of clusters using gap statistic.


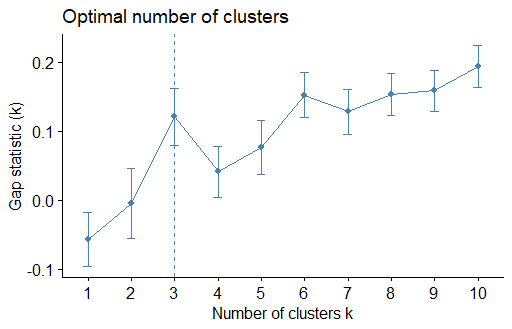


**Figure S3b.** Identification of clusters using elbow method.


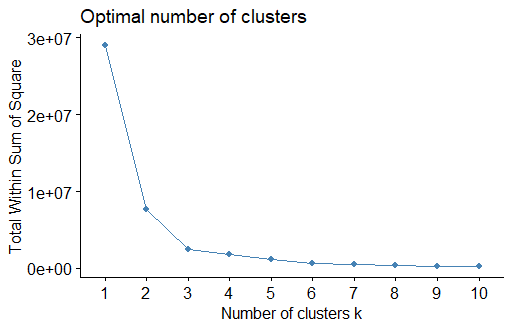


**Table 3.** Clusters Identified (k=3).

| Cluster 1  (“Typical Engagers”) N, % | Cluster 2  (“Early Engagers”) N, % | Cluster 3  (“Efficient Engagers”) N, % |
| --- | --- | --- |
| 81/202 (40%) | 58/202 (29%) | 63/202 (31%) |

Sensitivity Model 3: This model included only baseline and EOT (8 week) app utilization metrics, instead of including them for all 8 weeks. The purpose of this was to “weight” app utilization metrics more similarly to therapeutic alliance measures, which were only included at day 3 and EOT to see if this affected the pattern of results.

**Figure S4a.** Identification of clusters using gap statistic.


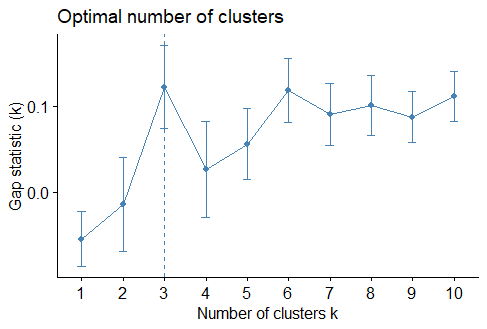


**Figure S4b.** Identification of clusters using elbow method.


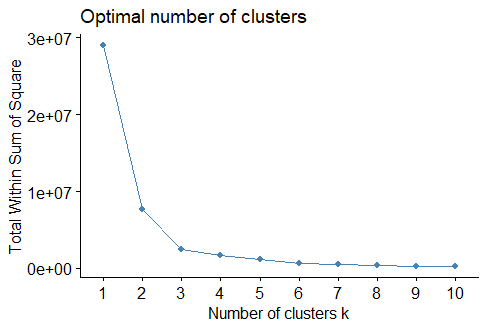


**Table 4.** Clusters Identified (k=3).

| Cluster 1  (“Typical Engagers”) N, % | Cluster 2  (“Early Engagers”) N, % | Cluster 3  (“Efficient Engagers”) N, % |
| --- | --- | --- |
| 81/202 (40%) | 58/202 (29%) | 63/202 (31%) |
